# Delivering remote pulmonary rehabilitation in Bangladesh: a mixed-method feasibility study

**DOI:** 10.7189/jogh.15.04002

**Published:** 2025-02-14

**Authors:** GM Monsur Habib, Nazim Uzzaman, Roberto Rabinovich, Sumaiya Akhter, Mustari Sultana, Mohsin Ali, Hilary Pinnock, RESPIRE Collaboration

**Affiliations:** 1 Community Respiratory Centre, Khulna, Bangladesh Primary Care Respiratory Society (BPCRS), Khulna, Bangladesh; 2 Centre for Inflammation Research, QMRI, The University of Edinburgh and Respiratory Department, Borders General Hospital, Scotland, UK; 3 NIHR Global Health Research Unit on Respiratory Health (RESPIRE), Usher Institute, University of Edinburgh, Edinburgh, UK

## Abstract

**Background:**

Pulmonary rehabilitation (PR) is an effective and essential component of care for the increasing number of individuals with chronic respiratory diseases (CRDs). Despite the benefits, it remains underutilised and poorly accessible in low- and middle-income countries (LMICs). We aimed to determine the feasibility of delivering PR in Bangladesh at home because of pandemic travel restrictions.

**Methods:**

Aligned with the Medical Research Council framework of development and evaluation of complex interventions, we recruited individuals with CRDs from the Community Respiratory Centre, Khulna, to a mixed-methods feasibility study. We assessed their functional exercise capacity and quality of life before and after an eight-week course of home PR, and conducted semi-structured interviews with PR providers and professional stakeholders by using a topic guide aligned with the normalisation process theory (NPT) and interpreting the findings within its constructs.

**Results:**

We recruited 51 out of 61 referred patients with a range of CRDs, of whom 44 (86%) completed ≥70% of their home PR course. Functional exercise capacity, measured by the endurance shuttle walk test, improved in 78% of patients, with 48% exceeding the minimum clinically important difference (MCID). Health-related quality of life, measured by the Chronic Obstructive Pulmonary Disease Assessment Test, improved by more than the MCID in 83% of patients. Through the interviews, we found that PR providers encountered challenges in remote video supervision due to unstable internet connections, forcing them to resort to telephone calls. The strength of support for NPT constructs varied; many participants understood and appreciated the role of PR and could make sense of the innovation (NPT-1), and most were assessing the potential of a PR service in Bangladesh to decide if it was worthwhile (NPT-4). Participants were not yet ready to endorse or actively support (NPT-2) or operationalise (NPT-3) the roll-out of PR.

**Conclusions:**

A home PR programme, supported by remote supervision and monitoring, is feasible in Bangladesh, but local evidence will be needed to promote implementation.

Chronic respiratory diseases (CRDs) play a significant role in causing morbidity and mortality, disproportionately burdening low- and middle-income countries (LMICs) [1,2]. Among these CRDs, chronic obstructive pulmonary disease (COPD) stands out as the third leading cause of death worldwide. LMICs, including Bangladesh, bear the heaviest burden of COPD and are experiencing increases in its prevalence [3]. Various factors such as high smoking rates, tobacco cultivation, exposure during tobacco processing, respiratory infections (including pulmonary tuberculosis), and poor air quality in workplaces all contribute to this growing burden of CRDs [2].

Pharmacotherapy can improve pulmonary function, alleviate symptoms, and reduce exacerbations, but does not break the cycle of decline associated with breathlessness, inactivity, and de-conditioning [4], punctuated by exacerbations that sometimes lead to hospitalisations. There is robust evidence, mainly from high-income settings, that pulmonary rehabilitation (PR), a personalised exercise programme based on individual assessment of exercise capacity and containing educational and other evidence-based components, can effectively address this cycle [5] and reduce the burden of COPD and other CRDs [6–9]. It is a comprehensive, multifaceted approach that demands the involvement of a team of healthcare professionals. In LMICs, the provision of such structured PR services is scarce [10], though chest physiotherapy focussed on postural drainage sometimes with advice on maintaining physical activity is widely available. The feasibility, delivery, and implementation of PR in Bangladesh have not yet been evaluated.

Our recent systematic review of 13 heterogeneous studies (11 at high risk of bias) from seven LMICs [11] concluded that multicomponent PR programmes can be delivered in low-resource settings and were associated with improvement in functional exercise capacity, health-related quality of life, and breathlessness. The review pointed to the need for PR services that are effective across a broad range of (potentially poorly differentiated) CRDs, overcoming barriers of cost, distance, and access to healthcare such that they are deliverable and sustainable in low-resource settings. Stakeholder engagement events in Bangladesh identified key implementation challenges [12] which informed our adaptation of a PR service that could be potentially deliverable in Bangladesh.

In this mixed-method study, we aimed to assess the feasibility of implementing PR in Bangladesh. Specifically, we sought to gather insights into the ideal resource infrastructure, strategies for delivering the components of PR, and to understand the challenges and facilitators of implementation as perceived by healthcare professionals, PR providers, and policymakers.

## METHODS

Aligned with the Medical Research Council framework of development and evaluation of complex interventions, we conducted this mixed-method feasibility study from 2021 to 2022, following ethical approval from the Institutional Review Board of the BRAC University (reference: 2019-045-ER, date: 24 September 2020). The study was sponsored by the Academic and Clinical Central Office for Research and Development at the University of Edinburgh (reference: AC 200004, date: 14 September 2020).

### Setting and intervention

We conducted the research at the PR Centre of Community Respiratory Centre Khulna (CRCK), Bangladesh, which identifies patients eligible for PR, conducts baseline assessment, and creates individually-tailored PR packages including exercise programmes, educational sessions, and outcome measurements. The start of the study coincided with the onset of the coronavirus disease 2019 (COVID-19) pandemic, so we had to adapt the programme for home delivery in our low-resource setting [13].

Following centre-based assessment, we arranged home PR training sessions lasting 60–90 minutes, conducted twice a week at home for eight weeks remotely-supervised by PR therapy staff. During the supervisions, the staff checked the patients’ exercise performances and adapted their intensity accordingly. We encouraged the patients to perform self-supervised exercise sessions on the other days of the week. The home PR programme comprised warm-up exercises, endurance (walking, cycling, or other endurance activities), upper and lower limb stretching and strengthening, relaxation exercise and education (disease knowledge and medication, self-management skills such as inhaler technique and airway clearance, coping strategies, breathing techniques, and nutrition). Each patient received an educational and home exercise booklet. The assessment at the end of the PR course was also conducted at the centre.

### Participant recruitment to the PR programme

We recruited patients from the CRCK, which serves individuals from a wide range of areas (both nearby and distant) with diverse socioeconomic statuses. We used a purposive sampling approach to ensure a diverse group that could be representative of the broader population. After the clinicians had diagnosed the patient, optimised their pharmacotherapy, and formulated a management plan, a research assistant invited them to participate in the study and obtained their informed consent. Our inclusion criteria were broad: we enrolled clinically diagnosed CRDs patients aged ≥18 years, only excluding individuals who had recognised contraindications to undertaking PR or those unable to exercise due to severe arthritis or paralysis [14–16]. We did not perform a sample size calculation, but planned to enrol approximately 50 patients with diverse CRDs, considering it to be a sufficient number of participants to assess the feasibility of the adapted PR, and practical within the available resources [17].

### Quantitative data collection and analysis

We assessed the participants at baseline and at the end of the eight-week PR course, with the second assessment carried out by an assessor blinded to the baseline results. Our primary outcomes of interest were the patients’ scores on two tests:

− The Endurance Shuttle Walking Test (ESWT), a tailored assessment of exercise tolerance that is reproducible and responsive to change after PR in COPD and other CRDs, for which 174 seconds is considered the minimum clinically important difference (MCID) [18];

− The COPD Assessment Test (CAT), a validated test for the evaluation of the impact of COPD on health-related quality-of-life (HRQoL) [19] for which the MCID is 2 [20].

For our secondary outcome, we assessed the patients in: three ways:

− Using the Modified Medical Research Council (mMRC) dyspnoea scale, which assesses the degree of breathlessness due to activity [21] and for which the MCID is 0.5 [22];

− Using the Hospital Anxiety and Depression Scale (HADS), with the MCID of HADS anxiety and depression scores being 1.32 and 1.40, respectively [23];

− By keeping records of PR attendance and completion rates (defined as at least 70% of the sessions recorded in the exercise diary) [24].

Our study was designed to demonstrate the intervention’s feasibility and was not powered to demonstrate an effect. We present before and after descriptive statistics alongside individual changes relative to the MCID; however, to avoid over-interpretation, we did not undertake any inferential statistics.

### Recruitment of professionals for qualitative interviews

We recruited a purposive sample of PR providers, healthcare professionals (HCPs), and policymakers. The HCPs involved in delivering the PR exercise (including the therapists in the PR team) were interviewed by NU, an experienced qualitative researcher who is not connected with the PR Centre and thus has the freedom to express their opinions. Interviewees were advised that the objective of our study was to learn about and improve the PR service so that critical comments and suggestions would be welcomed. We also interviewed other stakeholders whose opinions and perceptions could facilitate or block the future implementation of PR in Bangladesh. We anticipated that this would require up to 15 interviews with professionals, health service managers, and other stakeholders.

### Qualitative data collection

We conducted one-to-one interviews (face-to-face at the professional’s place of work) with the members of the PR provider team from the CRCK and from an associated PR centre in Dhaka. We interviewed the health service managers, policymakers, and other stakeholders over the telephone and arranged additional interviews with professionals attending in-person post-graduate training seminars. The interviews were conducted in Bengali and lasted about 30–45 minutes.

We used normalisation process theory (NPT) to inform the topic guide (**Table 1**; Table S1 in the **Online Supplementary Document**) [25–28]. This framework allows a comprehensive exploration of perceptions related to implementation and provides an opportunity to create meaning relevant to the implementation process [28]. It is not a scoring system; rather it aims to stimulate discussion and reach consensus on the normalisation potential of new techniques and technologies in healthcare settings [29]. We adapted interviews to the expertise of the interviewee. For example, in interviews with some HCPs, the NPT online tool kit was used to stimulate discussion, whilst interviews with therapists focussed on practical aspects of the delivery of remote PR in their setting.

**Table 1:** Topic guide informed by the constructs of NPT

Constructs of NPT (description)	Questions adapted to the PR intervention in Bangladesh
Coherence (how participants understand and value PR)	Can you distinguish between PR and the current therapy approach?
	Is PR comprehensible to the team for implementation in clinical practice?
	Is it feasible for the provider to deliver specific components of PR?
	Do you believe that PR can be beneficial for individuals with CRDs?
Cognitive participation (how participants buy-in and support PR in practice)	Can PR be effectively managed by designated individuals in a new setting?
	Can PR be executed collectively by different assigned individuals?
	Can various assigned members effectively carry out their respective tasks?
	Is it possible to make modifications once they have commenced their work?
Collective action (the operational work required for PR implementation)	Can the patient and provider collaborate effectively to implement PR?
	Are there skilled personnel readily available for PR in Bangladesh?
	Is it feasible to distribute various PR tasks successfully among the team?
	Can the existing PR resources be allocated to the team?
Reflexive monitoring (how participants judge whether PR is worthwhile)	How do you believe PR can be most impactful in clinical practice?
	Do you think PR can effectively function regardless of the specific setting or context?
	Can PR be a valuable tool in building relationships with health care providers?
	Do you think PR can be customised to meet specific needs?

CRD – chronic respiratory disease, NPT – normalisation process theory, PR – pulmonary rehabilitation

### Qualitative analysis

We transcribed the digitally audio-recorded interviews verbatim and coded them in Bengali to avoid losing nuance in translation. We then translated the transcripts and codes into English for sharing with English-speaking co-authors and for publication. The NPT constructs, interpreted with the example of the NPT coding manual [30], formed the basis of the coding framework, though we remained open to additional and unexpected themes emerging from the data.

### Mixed-method synthesis of data using the NPT toolkit

**Figure 1:**
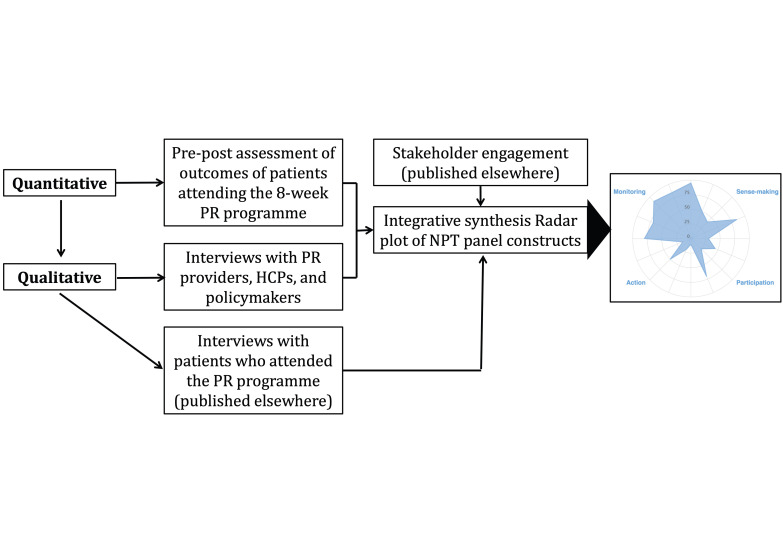
Overview of the mixed method study. HCP – health care provider, PR – pulmonary rehabilitation.

We synthesised the findings from both quantitative and qualitative data to address our objectives [31–33] and conducted a side-by-side comparative analysis (**Figure 1**), whereby the qualitative data provided context and validation for the quantitative findings [34].

We arranged a panel discussion including several of the study authors (HP, RR, MH, NU, SA, MS, and MA) to create a radar plot of the NPT constructs that indicated areas of strengths and weaknesses of the implementation of PR in Bangladesh [35].

The panel were informed primarily by the perceptions of the professional/stakeholder participants and the quantitative findings from the PR programme, but they were also aware of our previously published stakeholder engagement work [12] and views from the patient participant interviews [36]. The panel discussed each of the constructs in light of these data. Once agreed with all team members, the strength of each construct (from ‘very low’ to ‘full’) was assigned on the sliding bar of the online NPT toolkit, producing a radar plot [37]. This consensus process facilitated the understanding of the implementation of PR in Bangladesh and the formulation of implications and recommendations.

### Trustworthiness, reflexivity, and managing conflicts of interest

We considered the trustworthiness of our findings [38,39], considering that the researchers conducting the study had prolonged engagement with diverse aspects of respiratory care in Bangladesh specifically in the delivery of PR in LMICs. The first author (MH) is the senior physician and owner of the CRCK and is well-known in Bangladesh for promoting respiratory care, including PR. Another researcher (NU) conducted the interviews with therapists employed by the centre, and the transcripts from these interviews were anonymised before being given to MH for initial coding so he could not anticipate or misinterpret statements from participants he was personally acquainted with. The codes and the NPT Toolkit synthesis were then discussed with a multidisciplinary group unconnected with the PR practice to ensure a balanced interpretation, while the preliminary findings were discussed critically with colleagues from the National Institute for Health and Care Research (NIHR) Global Health Research Unit on Respiratory Health who are developing care for noncommunicable diseases in PR in Bangladesh, India, and Malaysia [40].

Our mixed methods approach enabled triangulation, enhanced by an NPT workshop which enabled multidisciplinary interpretation of all findings from the work. We generated the codes from the anonymised transcripts of interviews with a pragmatic and constructive approach constantly reminding ourselves to be open to all possible emerging data or themes (whether positive or negative). Finally, we have provided a structured description of the PR (Table S2 in the **Online Supplementary Document**) to enable readers to assess transferability.

## RESULTS

### Recruitment and baseline characteristics

We recruited 51 out of the 61 eligible patients referred from the clinic, of whom 33 (65%) were males and 18 (35%) females aged between 20 and 90 years with a range of CRDs, including COPD (41%), asthma (27%), pulmonary impairment after tuberculosis (10%), interstitial lung disease (6%) and asthma and COPD overlap (16%). The participants came from various professional, geographical, educational, and socioeconomic backgrounds (Table S3 in the **Online Supplementary Document**)

### Quantitative findings

#### Uptake and completion of PR

All 51 participants completed the baseline assessment and underwent the initial face-to-face PR session during the same visit to the CRCK. Each patient was offered a further 15 remotely-supervised sessions; 44 (86%) completed ≥70% (11 out of 16) of their home PR course. At the end of the eight weeks, 40 (78%) participants attended the post-PR assessment. Unfortunately, eight participants could not travel to the CRCK due to COVID-19 restrictions and concerns about the risk of infection.

#### Primary outcomes

**Figure 2:**
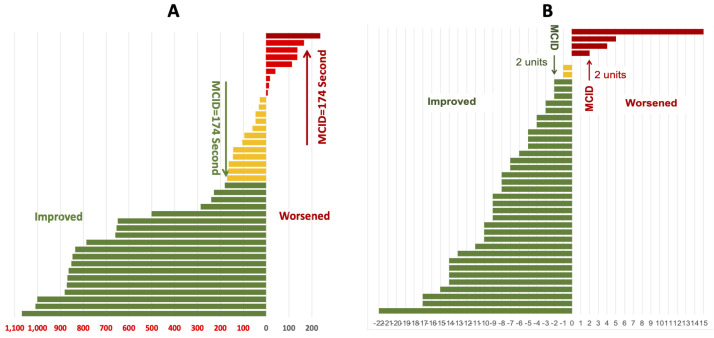
Primary and secondary outcomes. **Panel A.** Individual changes in functional exercise capacity related to MCID of the ESWT in seconds. **Panel B.** Individual changes in quality of life related to MCID of the CAT score. MCID – minimum clinically important difference.

Of the 40 patients who attended the post-PR assessment, 31 (77.5%) demonstrated improvement and 9 (22.5%) experienced a decline in their functional exercise capacity, as measured by ESWT in seconds. Almost half (n = 19, 47.5%) showed improvement greater than the MCID, while only one (2.5%) experienced a decline greater than the MCID (**Figure 2**, Panel A). Similarly, out of 40 patients, 33 (83%) improved their HRQoL by more than the MCID of the CAT score, and four (10%) deteriorated by more than the MCID (**Figure 2**, Panel B).

Regarding the change in secondary outcomes in the 40 patients who completed the post-PR assessment (Figure S1–3 in the **Online Supplementary Document**), we found that breathlessness, measured by the mMRC, improved in 25 (62.5%) and deteriorated in three (7.5%) patients by more than the MCID; anxiety, measured by HADS-A, improved in 16 (40%) and deteriorated in nine (22.5%) patients by more than the MCID; and depression, measured by HADS-D, improved in 27 (68%) and deteriorated in six (15%) patients by more than MCID.

### Qualitative findings

Sixteen professionals contributed to interviews (seven HCPs, four PR providers, two general practitioners (GPs)/owners of PR centres, and three nurses) (Table S4 in the **Online Supplementary Document**). They discussed their experience of delivering PR and its impact on patient care. Most providers considered that patients achieved better quality of life and independence due to the beneficial effect of PR. However, providers faced difficulties in the remote supervision of PR sessions due to the poor telecommunication network.

Some expressed concerns that patients on home PR programmes were less receptive to supervision and less adherent than those who attended at a centre. One participant stated that ‘Patients with the home PR are reluctant to accept any supervision over the telephone. They found it useless. ‘Nothing is new compared to what we learned at the centre” stated one patient when I called him for supervision of home exercise training’ (PR provider 1, therapist).

Patient motivation varied, with particular concerns that ‘those who are pushed to come here by their sons, daughters or other family members, do not improve well’; in contrast, ‘those who are sincere and want to keep themselves fit get better results’ (PR provider, pulmonologist).

Delivering home PR faced practical challenges, such as unstable internet connections and difficulty communicating with some elderly people. These issues necessitated changes to the original plans, including switching from video calls to audio calls and involving a family member to supervise the exercise program at home, guided by the therapist at the centre. The therapists recognised these strategies as viable alternatives for dealing with poor remote connections. One participant said that ‘(…) in our home PR programme, we planned to supervise the exercise training over a video call, however, because of the frequent disruption of the call due to poor internet connection we modified the intervention to an audio call to continue the supervision’ (PR provider 3, therapist). Another noted how ‘(…) those who are comparatively older, I used to ask them if anyone was staying nearby and could take the accompanied person’s help to make the discussion easier’ (PR provider 2, therapist).

#### NPT construct 1: Coherence (how participants understand and value PR)

PR is a new intervention in Bangladesh and, although most interviewees understood that PR could help people with CRDs to stay stable and active, few had any personal experience. Lack of awareness amongst HCPs was highlighted as a barrier to implementation, exacerbated by confusion with the familiar ‘chest physiotherapy’ (airway clearance techniques only). As a participant stated, 'It [PR] is completely a different topic from what I learned from medical college’ (internist, cardiopulmonary rehabilitation). Participants already delivering PR reported their understanding of how PR could fit into the Bangladeshi context, but other HCPs responsible for referring to services wanted more locally relevant evidence. One participant stated that ‘(…) we have no evidence on PR, I want to observe a few examples before referring the patients to the PR service’ (GP 1). Others also mentioned the financial burden to patients of adding one more item to the cost of CRDs management was also mentioned.

#### NPT construct II: Cognitive participation (how participants buy in and support PR in practice)

Among our participants, PR was seen as a patient-centred complex intervention delivered by a multidisciplinary team, but the lack of institutional training programmes meant services in Bangladesh relied on in-house training by a few primary care physicians with specialist expertise. The lack of ‘skilled manpower’ meant that even in specialist hospitals that had a ‘chest physiotherapist’, activities were limited to airway clearance techniques.

Most of the participants emphasised the need for teamwork, including pulmonologists, primary care physicians, PR-trained assistants, and other relevant healthcare providers, with one participant stating that ‘A “one-man show” is not appropriate for implementing PR in Bangladesh’ (HCP 1).

One practical systems-level barrier was the concern that a referral could result in the patient moving permanently to the PR practice and losing income for the referrer. As a HCP said, ‘We could not yet develop a referral system in our practice; once I refer my patient to a PR centre, the patient might have a good impression of the PR-providing physician and may not come back to me, leading to loss of the patient from my practice’ (HCP 3).

#### NPT construct III: Collective action (the operational work required for PR implementation)

Most of the practitioners would like to integrate PR into their practice, but saw major challenges (*e.g.* no financial recompense) that made it difficult to accomplish. A participant noted that ‘Adding PR in my current practice will enrich the service of the patient and I find no challenges except an extra payment for the PR service (…)’ (PR provider 1, therapist).

Geography and long travel times were recognised as barriers and one centre had already initiated an on-site ‘express service’ offering a week-long intensive exercise therapy after which the patient continued the programme at home with telephone support.

Staff in the three established PR centres in Bangladesh worked as teams with mutual trust and respect in terms of their roles and performance. Physicians were involved in diagnosis, motivation, referral, and overseeing the programme. Medical assistants were trained to undertake assessments, education, and record-keeping, and trained nurses supervised exercise training. A PR provider (pulmonologist) commented that ‘In our centre, four people are working in the PR unit, two qualified and PR-trained medical assistants, one nurse, and an informally trained exercise trainer. Although we allocated our tasks according to knowledge, skill, and interest, we work together in difficult cases and assist each other when needed.’

The scarcity of PR-trained and skilled personnel in Bangladesh was a challenge, and most training was in-house and provided within the centres. One participant noted that ‘I have received training from sir [lead physician] in our practice setting, which has involved working with several patients since the beginning of the centre's establishment’ (PR provider 1, therapist). A PR provider (pulmonologist) recalled that ‘As a PR therapist, I have provided PR for about 600 patients and learned a lot by practice although I don’t have institutional training’.

‘Chest physiotherapists’ had been appointed in specialist hospitals, but poor understanding of the distinction between chest clearance and PR, alongside competing demands on the limited budget, was implicated in the lack of policy-level support for the implementation of PR.

#### NPT construct IV: Reflexive monitoring (how participants judge whether PR is worthwhile)

Interviewees who were providing PR services described how they used the pre-post assessments to audit the effectiveness of their programme, with one provider describing monthly meetings for the evaluation of their performance which they described as ‘encouraging’. Providers considered that implementation of PR not only benefitted patients by reducing the burden of living with CRDs but also added value for the practice, and some felt could potentially improve respiratory care at a population level. One GP said that ‘When conventionally we are providing a prescription for pharmacotherapy and expecting a good result, it is not the fact. A trained and skilled PR team can change the whole scenario of CRDs care in Bangladesh, I believe (…)’. A PR provider (pulmonologist’ commented how ‘Patients are more satisfied with our service than ever. It’s a pleasure for us, also our workload on a specific patient is remarkably reduced.’

A point for discussion for several interviewees was having an appropriate referral pathway, which ensured that other causes for breathlessness were excluded and that PR was recommended at the optimal stage in the patient’s illness. For example, an internist of cardiopulmonary rehabilitation noted how ‘Pulmonologists are diagnosing frequently dilated cardiomyopathy as COPD, not doing an echocardiography; prescribing a few inhalers and that’s why PR is not working well.’

#### Over-arching synthesis and assigning strength to the NPT constructs

**Figure 3:**
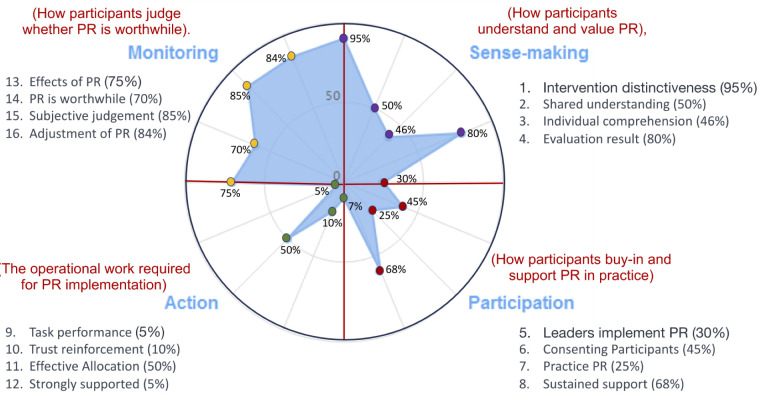
Radar plot from NPT constructs and 16 variables. Positive responses extend further out from the centre than negative ones, ie, the more positive a response, the further it extends from the centre with more negative responses nearer to the centre (‘0’). PR – pulmonary rehabilitation.

While a few local centres pioneered multidisciplinary PR services, most professionals and stakeholders focussed on evaluation through informal monitoring and formal research and on raising awareness of PR. Although there was some sense-making and sporadic participation, these efforts did not translate into meaningful action. Key barriers included a lack of policy support, confusion with chest physiotherapy services, unclear referral mechanisms, and the absence of official training or career pathways for PR staff (**Figure 3**, **Table 2**; Table S5 in the **Online Supplementary Document**).

**Table 2:** Development of constructs of NPT

No.	Component	Assigning the strength of the component	Exemplar quote
**Construct 1: Coherence (Sense-making – how participants understand and value PR)**			
1	Differentiation	All participants recognised PR as a novel approach which was different to current practice. – Nearly full strength	It [PR] is completely a different topic (…) Adding to our basic medical knowledge this PR technique will certainly help the patients a lot. – Internist; cardiopulmonary rehabilitation
2	Communal specification	Most participants understood and considered PR as potentially effective and useful, but many of them had no practical experiences, relying mainly on theoretical perception. – Moderate strength	(…) but I am not sure, it will work for our patients with ILD and post-TB lung fibrosis.—, I want to observe a few examples before referring the patients to the PR service. – GP 1
3	Individual specification	Only a few PR centres are operating currently in Bangladesh. Within these centres the role of staff is clear, but in general, the role of other stakeholders such as health care professionals, and health care planners is yet to develop. – Low-moderate strength	We need to tell people first, what COPD is (…) for example, smoking is the main reason; now if you don’t stop smoking and go to rehab, it will illogical and redundant. – GP, respiratory medicine
4	Internalisation	In general, the concept was understood and PR was considered as a useful and effective intervention once implemented and integrated in a practice. – Near-full strength	It is observed that in comparison to the baseline parameters, post-intervention parameters are significantly improved (…). – PR provider pulmonologist
**Construct II: Cognitive participation (the relational work that people do to build and sustain a new practice)**			
5	Initiation	Most participants are listening, watching and observing PR as a new intervention, they hadn’t yet decided to ‘buy-in’ the service in their own practice; accordingly, they are not helping to drive the implementation process of PR. **–** Low strength	Basically, we don’t have any institutional training programme on PR (…). – PR provider pulmonologist
6	Legitimation	Most participants lacked prior involvement in PR but a few offered PR services in Bangladesh, with limited experience, yet they hoped to contribute post-information acquisition. – Upper end of low strength	(…) they conduct chest physiotherapy with a few manoeuvres like patting for chest clearance (…), but they don’t bother about PR. – Health care professional 3
7	Enrolment	The majority are observing PR process progress in Bangladesh, but they haven't committed to joining yet, making their readiness for this new initiative unclear. – Low strength	(…) so, you need to engage all levels of HCPs under the leadership of a pulmonologist/chest physician. – Health care professional 1
8	Activation	After PR implementation, willing participants agreed to identify actions for sustainability and stay involved. – Moderate strength	(…) we arrange two motivation classes (…). All the team including doctors, medical assistances, therapists etc., counsel them. – PR provider, pulmonologist
**Construct III: Collective action (the operational work that people do to enact a new practice)**			
9	Interactional workability	Many interviewees were unaware of operationalising PR, including its components, and lacked experience in implementing the intervention. – Very low strength	I read about it in textbooks only (…). It is now considered an integral part of chronic respiratory care. – Health care professional 2
10	Relational integration	Participants theoretically trusted the intervention, but real-life outcomes remained uncertain. Lack of prior PR collaboration led to hesitancy in mutual trust. – Low strength	Our responsibility is distributed according to the interest of the team members, and we are doing well, developing skills (..) an excellent workup, we founded so far (…). – PR provider pulmonologist
11	Skill-set workability	Limited multidisciplinary staff exist, but overall, interest hasn't translated into action in Bangladesh. Nationally, trust issues surround varied roles, like therapists lacking formal training. – Moderate strength	(…) You need such dedicated, trained, and skilled manpower (…). We don’t have trained manpower for PR. – Internist, cardiopulmonary rehabilitation
12	Contextual integration	Nationally, no policy supports PR in planning, funding, or resources. In Bangladesh's health sector, government, private sector, NGOs, and partners lack motivation for this health care intervention. – Very low strength	In our centre, four people are working in the PR unit with only two informally PR-trained medical assistants (…). No national priority is taken so far. – PR provider pulmonologist
**Construct IV: Reflex monitoring (the appraisal works that people do to assess and understand how a new practice affects them and others)**			
13	Systemisation	Despite a lack of practical experience, most participants expressed their confidence in determining the effectiveness and usefulness of PR by formal or informal methods. – Moderately full strength	Certainly, it is effective. We measure the outcome after 8 weeks of therapy and find improvement in all the parameters. – PR provider 3
14	Communal appraisal	PR centres show good monitoring and feedback. Participants gauge intervention effects. Yet, national activities mostly focus on monitoring. – Mildly full strength	PR is very important and effective for the care of CRDs. I think PR is not an optional but integral part of CRD management (…), I believe (…). – GP respiratory physician.
15	Individual appraisal	As described above, individuals engaged in providing PR are experiencing the impact and expressed their perception positively. – Highly full strength	However, you see, we are training the patients how to (…). Similarly, if a trained therapist shows the limb exercises (…). – A pulmonologist
16	Reconfiguration	PR mainly involves monitoring (research, dissemination, engagement) to raise awareness and interest, but with limited action. Participants anticipate eventual positive change. – Highly full strength	(…) in our home-PR programme, we planned to supervise the exercise training over a video call, however (…) an audio call works for supervision. – PR provider 3

COPD – chronic obstructive pulmonary disease, CRD – chronic respiratory disease, GP – general practitioner, ILD – interstitial lung disease, NGO – non-governmental organisation, PR – pulmonary rehabilitation, TB – tuberculosis

## DISCUSSION

### Summary of key findings

We demonstrated that a PR programme, adapted from global guidelines, is applicable and feasible in Bangladesh. Our home PR model, delivered during the COVID-19 pandemic, had an uptake of 84% and a completion rate of 86%. Participation in the programme was associated with improvements for most participants in exercise capacity, HRQoL, symptoms, anxiety, and depression. Simultaneously, PR providers considered PR a novel intervention in Bangladesh, with numerous challenges in its deployment and integration into standard clinical practice. Nevertheless, there was optimism that, if these hurdles could be surmounted, PR has the potential to improve the management of CRDs. Apart from staff in the three pioneering multidisciplinary PR centres in Bangladesh, the predominant activity amongst professionals and other stakeholders was evaluation (informal monitoring and formal research) and promoting the concept. There was evidence of sense-making among stakeholders and sporadic participation, but no translation into action. The obstacles include a lack of policy support, ambiguity with existing chest physiotherapy services, a lack of clarity regarding referral mechanisms, and the absence of official training and career pathways for PR personnel.

### Strengths and limitations

We undertook a mixed-methods study incorporating a quantitative assessment of outcomes and qualitative interviews with professionals and other stakeholders. These perspectives were complemented by patient views on living with CRDs and their perceptions of PR (reported elsewhere [36]), providing a multi-perspective overview of the PR service. The outcomes we selected (functional exercise capacity, HRQoL, breathlessness) not only reflect core outcomes as recommended by the Core Outcome Measures in Effectiveness Trials (COMET) [41,42], but proved to be feasible to use in the low resource setting and with the population served by the CRCK, including the Bengali language translation of the questionnaires (Table S6 in the **Online Supplementary Document**).

Most participants improved by more than the MCID, but no conclusions should be drawn about effectiveness, given that this was a feasibility study and that we did not perform a sample size calculation on our primary outcomes. Our ‘before and after’ design did not have a control group, and the improvements may have been due to the recognised benefits associated with participating in a research study. Nevertheless, the improvements were substantial and consistent across all the outcomes, suggesting that a fully-powered randomised controlled trial of PR delivered in low-resource settings to people with (potentially undifferentiated) CRDs is warranted. Subsequently, a three-arm, individually randomised hybrid-1 implementation trial (the PuRe trial) commenced in March 2024 and will assess the clinical and cost-effectiveness of PR delivered in four LMIC settings [43].

We have been explicit that MH is a provider of PR and the owner of the CRCK, which is a respiratory-only primary care practice in Bangladesh. Our practice of reflexivity and the involvement of multidisciplinary teams in interpretation enabled us to remain aware and reduce the impact of this conflict of interest. Although the study took place in 2021, preparation commenced before the COVID-19 pandemic, so the participating team had ample opportunities to review their processes, and develop, implement, and standardise PR practices within their context.

The strict movement restrictions implemented during the COVID-19 pandemic may have influenced the shape of CRDs among the participants, as the majority of attendees at the clinic were patients who came in for an unscheduled visit due to a worsening of their condition. Pandemic-related travel restrictions made it challenging for patients to visit the centre for outcome assessments (as opposed to undertaking routine care), increasing attrition. Furthermore, they were reluctant to spend extended periods in a clinical setting. Additionally, most of the interviews were conducted remotely, and we had difficulties in a few interviews due to the poor telecommunication network.

### Discussion in relation to published literature

The World Health Organization's ‘Rehabilitation 2030: Call to Action’ emphasises the pivotal role of accessible and affordable rehabilitation and recognises a significant unmet need, particularly in LMICs, where the demand for pulmonary rehabilitation far surpasses the available capacity [44]. Despite some international initiatives promoting PR services in LMICs [45], there remains a need for a systematic approach to adapting and implementing PR in low-resource settings. To address this issue, we have conducted a systematic review [11] and a series of stakeholder meetings to understand the context of Bangladesh for implementing PR [12], and have adapted PR guidelines from global sources to make them applicable and feasible for delivery in Bangladesh [13]. Here we have shown that implementing PR for people with CRDs and measuring outcomes is feasible in low-resource settings [11,46].

Different PR models have been evaluated in LMICs, including centre-based, home-based, or community-based programmes [11], each with its own advantages and disadvantages. Given the accessibility challenge, some patients prefer home-based programmes (*i.e.* home PR), while others favour centre-based ones [11]. Patients facing travel difficulties, meanwhile, often opt for community-based programmes [13]. The combination of a pandemic and technological advances has promoted the use of remotely delivered PR [47-49], though access to digital options was challenging for some patients and the therapists had to be flexible to find options that worked for everyone. In our study, we provided remote supervision of home PR, necessitating substantial modification of our usual practice to deliver an optimal service.

We identified several key messages in our qualitative analysis which focussed on the importance of engaging stakeholders, raising awareness, addressing training needs, and garnering political support. Despite some confusion with ‘chest physio’ and/or ‘airway clearance techniques’, HCPs recognised PR as a distinct intervention compared to current management strategies of CRDs and agreed that it was feasible in LMICs, which aligns with findings from Uganda [46]. A few professional participants in Bangladesh have been involved in delivering PR for several years and are optimistic about its potential but desire further learning. In a clinical review, Lahham et al. [50] describe similar challenges in PR delivery in remote/rural areas and LMICs. Our HCPs highlighted the need for more practical information and awareness to engage effectively in PR. Similarly, the NIHR Global Health Research Group on Respiratory Rehabilitation (RECHARGE) standard international data set for PR in LMICs [51] echoed the importance of addressing these challenges. Stakeholders expressed a need for additional time to understand and evaluate PR and how it should be integrated into clinical practice. The Free Respiratory Evaluation and Smoke-exposure reduction by primary Health cAre Integrated gRoups (FRESH AIR) systematic review [52] of implementation strategies relevant to lung health in LMICs recommended understanding the needs of local users, tailoring to local contexts, engaging influential stakeholders, ensuring access to knowledge and information, and addressing resource availability to enhance implementation success and ultimately improving health outcomes. While many of these strategies are under way in Bangladesh, our analysis suggests that certain domains, such as participation and engagement (NPT constructs II and III), still require improvement for successful PR implementation.

The trends in attendance, completion rates, and uptake of PR in our study reflect global studies. Notably, the acceptance of home PR in our study is consistent with findings from other research [53], and participation in home PR supervision remained high, possibly reflecting the normalisation of remote medical consultations during the pandemic. Nevertheless, we identified consistent barriers to both participation in and completion of the PR programme, including limited transportation options for centre-based assessment of home PR, inconvenient scheduling, and disruption to daily routines. The home PR model emerged as a solution capable of overcoming these challenges, contributing to the observed high completion rates.

### Implications and recommendations

Our findings have some implications for healthcare services. First, we demonstrated that practical barriers to delivering remote pulmonary rehabilitation in a low-resource setting can be overcome, though this requires flexibility on the part of therapists. Second, we observed that ambiguity with existing chest physiotherapy services is a significant barrier which will require strategies to raise awareness and knowledge among practitioners. Third, we found that stakeholder engagement will be important to promote supportive policy changes; this could include better clarity regarding referral mechanisms and the provision of official training and career pathways for PR personnel. Lastly, we can conclude that there is a need for a robust, fully-powered trial including a health economic evaluation to demonstrate the benefits of pulmonary rehabilitation for people with CRDs in Bangladesh.

Based on these findings, we can draw recommendations for the successful implementations of PR in Bangladesh: we need to enhance institutional support, establish an effective referral mechanism, address the lack of formal training and career pathways, conduct health economic evaluations, and develop a delivery model for pulmonary rehabilitation that aligns with both the evidence from our context and the recommendations of stakeholders

## CONCLUSIONS

A PR programme adapted for our local setting in Bangladesh proved feasible and acceptable from our study. Home PR, essential in the pandemic, proved to be a viable and safe option that mitigated accessibility challenges in remote areas of Bangladesh. Qualitative interviews with professionals and stakeholders revealed a positive attitude to developing a PR service, but highlighted some remaining barriers. We hope that this study will inform the implementation of a comprehensive PR service in Bangladesh and other LMICs, thereby leading to improved health outcomes for patients with CRDs.

## Additional material


Online Supplementary Document
