# Supplementary material for: Delivering remote pulmonary rehabilitation in Bangladesh: a mixed-method feasibility study
Source: J Glob Health. 2025 Feb 14;15:04002. doi: 10.7189/jogh.15.04002 (PMC11825123; doi:10.7189/jogh.15.04002)

Supplement to: Habib GMM, Uzzaman N, Rabinovich R, Akhter S, Sultana M, Ali M, Pinnock H. Delivering remote pulmonary rehabilitation in Bangladesh: a mixed-method feasibility study. J Glob Health. 2025;15:04002.

**Contents**		
Table S1	Detailed topic guide for NPT interview	Page 2
Table S2	Components and mode of delivery of PR	Page 5
Table S3	Characteristics of Participants in Quantitative Study	Page 6
Table S4	Characteristics of Participants in Qualitative Study	Page 7
Table S5	NPT Panel members	Page 7
Table S6	TIDieR checklist for adapted PR programme	Page 8
Figure S1	Individual changes in mMRC	Page 10
Figure S2	Individual changes in HADS-A	Page 10
Figure S3	Individual changes in HADS-D	Page 11

Table S1: Detailed topic guide for NPT interview

Dimension	Outcome questions	NPT questions	Prompts
NPT Construct 1: Coherence (Sense-making - how participants understand and value PR)			
Differentiation	What has the PR changed in your current practice?<br>Is it worse or better than what you were doing before?	Do you think your practice is different now from what it was before PR?<br>What is the same, and what is different?	What has the PR programme changed?<br>For you?<br>For patients?<br>For different staff groups?
Individual specification	Does the programme make sense to people?<br>What do you understand the purpose of the SVP to be?<br>Has using the SVP helped you as an individual in providing continence care?	Do you think people understand the PR and what they have to do?	Are there any groups who have particular difficulties?<br>Senior staff?<br>HCPs?<br>Patients?
Communal specification	How did the team look after patients with CRDs before the implementation of PR?<br>Has using the PR helped the team in providing continence care?<br>Is the PR compatible with what you do?	Do you think people agree about the PR programme in terms of its:<br>Purpose?<br>How it works?	Do all stakeholders agree on what to do and why?<br>Qualified/non-qualified?<br>Nursing/medical?<br>Patient/relative?<br>Different wards?
Internalisation	What were your initial impressions of the programme?<br>Does it make sense to use the PR to look after patients with CRDs?<br>What do you think of the content of the PR?	Do you think people like the new programme, or not?<br>What are the costs and benefits of doing things this way? Would everyone agree?	Are there particular aspects that are liked more or less?<br>Praise?<br>Paperwork?<br>Support?
**NPT Construct II: Cognitive participation: the relational work that people do to build and sustain a new practice**			
Initiation<br>People drive the new practice forward	How did you find out about your unit's involvement in the PR?<br>Has there been enough direction for the programme?	Who are the key people driving the implementation of the programme?<br>Has this changed over time?	Who are the key people driving the implementation of the programme?<br>Has this changed over time?

Enrolment<br>Agreement on the new practice	How do you see your role with respect to the PR?<br>Do you see this as part of your role/someone else's role?<br>Has involvement been sufficient?	Do people think they should be doing this?<br>Is everyone on board, or are some people more involved than others?	Do people think they should be doing this?<br>Is everyone on board, or are some people more involved than others?
Legitimation<br>People buy in and organise themselves	How have the team worked together to change their practice?<br>How have the team organised themselves to deliver the PR?<br>Did anything get in the way of the programme working well?	Is the PR running smoothly now, or are there still glitches?<br>What sort of changes have you had to make to get the programme running smoothly on your ward?	Has it affected groups differently? For example:<br>Practice managers?<br>Qualified staff?<br>HCPs?
Activation<br>People work together to develop the new practice	What has helped staff introduce the PR?<br>How has the introduction of this PR compared with other practice development initiatives?	Has it affected how work is organised on the practice?<br>How?	What about how:<br>People are allocated to different areas of the practice?<br>Communication occurs?<br>The routine of the day works?<br>Night/day staff work?
NPT Construct III: **Collective action: the operational work that people do to enact a new practice**			
Interactional workability (How does the work get done?)	How did using the PR affect your interactions with patients?<br>How did using the PR affect your interactions with other staff?<br>How did you act to solve problems?<br>Is it realistic to do the PR on a day-to-day basis?	Can people do what is being asked of them?<br>Have there been any problems other than the ones you've mentioned (summarise)?	Can different groups do it?<br>Staff on all shifts?<br>All days of the week?<br>Patients?
Relational integration (Staff trust each other's work and expertise)	How did introducing the PR change the management of continence on the practice?<br>Have you noticed any other changes (or spin-offs) from the introduction of the PR on the practice?<br>Do you think everyone would feel confident that things are being done right?	Are you confident that the programme is being done as it should be?<br>By everyone?	Has it affected interactions between:<br>Staff and patients?<br>Qualified/HCP?<br>Different practice areas?<br>Different professions?<br>Day and night staff?
Skill set workability (How is the	How did using the PR affect 'who does what' in the management of CRDs?<br>What did you think about the training you got?	Is the work allocated to the right people?<br>Do people have the right skills and knowledge now? Is everyone trained up as much as they need to be?	Do different groups have the ability to do the PR programme?<br>HCPs?<br>Patients?

work distributed?)			
**Contextual integration (How is the work supported?)**	Are there resource implications of the PR?<br>Are there any other implications of using the PR?<br>Is there sufficient support and resources for the programme?	What sort of things have supported the implementation of the PR?	Time?<br>Money?<br>Staff?
NPT Construct IV: **Reflexive monitoring: the appraisal works that people do to assess and understand how a new practice affects them and others**			
Systematisation	How did you assess the value of the PR?<br>How does this fit with other systems in place to monitor and evaluate practice?<br>Is the programme working?	How do you know if the programme is working or not?<br>Do you know this for everyone?	What would success be for you? What would failure be?<br>Do you think this would be the same for the patients?
Communal appraisal	What factors might affect the decision of the team to support the PR?<br>What factors might affect the decision of the team to continue to use the PR when our project ends?<br>On the basis of what you have seen of its results, would the practice staff think it worth continuing?	Do you think people would agree about whether it works or not?	Practice managers?<br>All groups of staff?<br>Patients?
Individual appraisal	What factors might affect your decision to support the PR?<br>What factors might affect your decision to continue to use the PR when our project ends?	If it was up to you, would you carry on doing it?	What would affect your decision?
Reconfiguration	Do you think using the PR has affected the way clinical practice is organised?<br>How easy was it to implement?<br>If you could change one thing to improve the programme, what change would you make?	Do you think it is being done according to the instructions?<br>Has the programme been modified in any way to suit the ward, other than what you have already mentioned?	For better? For worse?<br>How compatible was it with other aspects of stroke patients' care?

Table S2: Components and mode of delivery of PR

Components of PR	Mode of delivery
Optimisation of pharmacotherapy	Pre-recruitment
Baseline and outcome assessment	Individual; Centre-based
Prescribed exercise based on assessment	Individual; Centre-based
Exercise training sessions	“Home-based”
Education (inhaler technique, advice on smoking/biomass, coping strategies, breathing techniques,) self-management,	“Remote (webinar, video-links)”
Individual smoking cessation support	“Remote (telephone)”
Psychological support	“Remote (telephone)”
Supervision	“Remote 8 x 2/week”
Group support	“Virtual groups”

Table S3 Characteristics of Participants in Quantitative Study

Identifier	Age	Sex	Diagnosis	Profession
FS 1	60-70	M	COPD	Jute mill worker
FS 2	20-30	F	Asthma	Housewife
FS 3	20-30	M	Bronchiectasis	Business
FS 4	60-70	M	COPD	Retired
FS 5	60-70	M	COPD	Journalist
FS 6	50-60	M	COPD	Business
FS 7	40-50	F	ACO	Housewife
FS 8	50-60	F	Asthma	Housewife
FS 9	60-70	M	Asthma	Retired teacher
FS 10	60-70	M	COPD	Retired teacher
FS 11	60-70	F	Asthma	Housewife
FS 12	50-60	F	Asthma	Housewife
FS 13	50-60	M	COPD	Meat seller
FS 14	50-60	M	COPD	Service holder
FS 15	60-70	M	ACO	Service holder
FS 16	60-70	F	Asthma	Housewife
FS 17	40-50	M	ACO	Business
FS 18	50-60	M	ILD	Housewife
FS 19	40-50	M	ILD	Business
FS 20	50-60	F	Asthma	Housewife
FS 21	60-70	F	COPD	Wood business
FS 22	50-60	F	Asthma	Housewife
FS 23	50-60	M	PIAT	Fish business
FS 24	40-50	F	Asthma	Housewife
FS 25	20-30	M	COPD	Business
FS 26	60-70	F	Asthma	Housewife
FS 27	50-60	F	PIAT	Housewife
FS 28	30-40	F	Asthma	Housewife
FS 29	50-60	M	COPD	Farmer
FS 30	50-60	M	ILD	Service holder
FS 31	80-90	M	COPD	Retired
FS 32	50-60	M	COPD	Retired
FS 33	70-80	M	COPD	Retired
FS 34	70-80	M	COPD	Retired
FS 35	60-70	M	COPD	Business
FS 36	60-70	F	ACO	Housewife
FS 37	50-60	F	Asthma	School teacher
FS 38	70-80	F	ACO	Housewife
FS 39	60-70	M	COPD	Ex jute mill worker
FS 40	50-60	M	PIAT	Ex jute mill worker
FS 41	50-60	F	Asthma	Housewife
FS 42	50-60	M	PIAT	Business
FS 43	30-40	F	Asthma	Housewife
FS 44	50-60	F	ACO	Housewife
FS 45	50-60	M	COPD	Service holder
FS 46	60-70	M	COPD	Ex-jute mill worker
FS 47	60-70	M	COPD	Retired
FS 48	50-60	M	ACO	Retired
FS 49	40-50	M	COPD	Farmer
FS 50	40-50	M	COPD	Business
FS 51	60-70	M	ACO	Business

Table S4: Characteristics of Participants in qualitative study

No.	Identifier	Setting	Care level	Occupation	Interview type
1	HCP 1	Urban	Tertiary care	Internist	Semi-structured
2	HCP 2	Urban	Tertiary care	Pulmonologist	Semi-structured
3	HCP 3	Urban	Primary care	Resp. physician	Semi-structured
4	HCP 4	Rural	Primary care	GP	Semi-structured
5	HCP 5	Urban	Tertiary care	Internist	NPT domains
6	HCP 6	Urban	Primary care	GP	NPT domains
7	HCP 7	Semi-urban	Mixed - care	GP	NPT domains
8	PRP 1	Urban	Primary care	Trained therapist	Semi-structured
9	PRP 2	Urban	Primary care	Trained therapist	Semi-structured
10	PRP 3	Urban	Primary care	Trained therapist	Semi-structured
11	PRP 4	Urban	Primary care	Trained physician	Semi-structured
12	PW 1	Urban	Tertiary care	Hospital owner	NPT domains
13	PW 2	Urban	Tertiary care	Hospital owner	NPT domains
14	SH 1	Semi-urban	Secondary	Nurse	Group interview
15	SH 2	Rural	Primary care	Nurse	Group interview
16	SH 3	Rural	Primary care	Nurse	Group interview

Table S5: NPT Panel members:

Members	Age	Sex	Affiliation
HP	60-70	Female	GP and Senior researcher, University of Edinburgh
RR	50-60	Male	Pulmonologist, International PR expert, UoE
MH	60-70	Male	GP, PhD (Pulmonary Rehabilitation) UoE
NU	40-50	Male	PhD Student, UoE
SA	20-30	Female	Medical assistant, PR provider at CRCK
MS	20-30	Female	Medical assistant, PR provider at CRCK
MA	20-30	Male	Medical assistant, PR provider at CRCK

Table S6: TIDieR checklist for adapted PR programme

Sr No	Item	How we conducted the Intervention in this study
1)	Brief name	Home-based PR for the patient with CRD
2)	Context	In Bangladesh, there is a growing prevalence of chronic CRDs. PR, which is crucial for CRDs management, has only been introduced in a few centres in Bangladesh recently. However, due to inadequate healthcare systems, financial constraints, and a shortage of trained personnel, PR remains underutilised. Both the general population and healthcare professionals have very little awareness of PR. As a result, implementing PR in our context presents substantial challenges.
2)	Why?	PR is an integral part of CRD care, it improves exercise tolerance, HRQoL, reducing the burden of CRD. Home-based delivery not only enables PR to be maintained during a pandemic, but also improves access as it enables flexibility and overcomes problems with travel.
3)	What?	Materials: protocols defined the process of assessing eligibility of patients, comprehensive assessment of needs, and individually tailored therapies. We provided exercise guidelines and handbooks for both the providers and patients respectively.
4)		Procedures: We have provided procedures for each of the activities, components and/or processes used in the intervention, including any enabling or supporting activities.
5)	Who provided?	Within available resources a small team worked together to provide the intervention. This was a senior respiratory referring physician, two medical assistants informally trained on PR, and a nurse. In addition, a non-clinical assistant works in the team occasionally.
6)	How?	Although originally, we proposed a face-to-face programme, due to COVID-19 it was changed to home-based PR, with initial and post-interventional assessment at the centre face-to-face.
7)	Where?	The centre was located at the community respiratory clinic, where the minimum essential equipment was available. The centre had access to a nearby secondary care hospital for any emergency event management.

8)	When and how much?	The programme was designed for 8 weeks with an initial assessment at the centre, then twice a week remotely supervised sessions of 45-60 minutes, and finally a post interventional assessment at the centre again Remote sessions were either supervised by video/telephone according to availability, especially for the first few sessions until the patients learned to do the exercise confidently. If connectivity was poor, a family member or friend could support the exercise at home. Moreover, the patient undertook unsupervised exercise at least 5 days a week for at least 40 minutes. The patients were not asked to keep a log of their exercise because of literacy problems,<br>The intensity was determined at the initial assessment session and thereafter tuned by ‘Talk Test’– described later.
9)	Tailoring	The intervention was tailored according to the baseline assessment. As it was a home-based programme, ‘FITT’ principle was used based on the availability, home-environment, and the indication for the intervention. It was titrated to the needs and ability of the patient
10)	Modifications	The intervention was modified according to need. For example, video supervision was switched to audio monitoring if internet connection was poor. Moreover, a witness or an assistant from family of the patient could be involved in the supervision programme (with due consent of the patients) when needed.
11)	How well?	Planned: Adherence/fidelity was an important element of the intervention from the perspective of the providers and patients. Fidelity of delivery could be assessed from the detailed logs of all contacts. Patient adherence was assessed in each supervised session from the reports of the providers, and by the patients’ written diary. Family members or friends could help with reporting activity if the patients were unable to keep a diary or memorise due to old age.
12)		Actual: We evaluated the fidelity of delivering patient education by assessing their knowledge on risk factor removal, inhalation technique, understanding of pharmacotherapy and regular exercise programme are also assessed by interviewing the patient in each session supplemented by the witness of family members.

Figure S1. Individual changes in mMRC

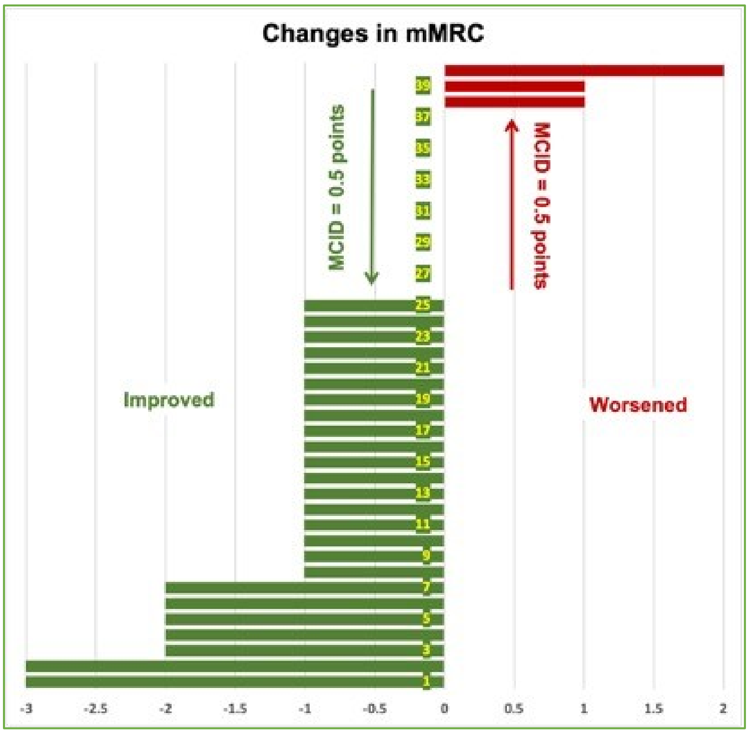

Figure S2: Individual changes in HADS-A

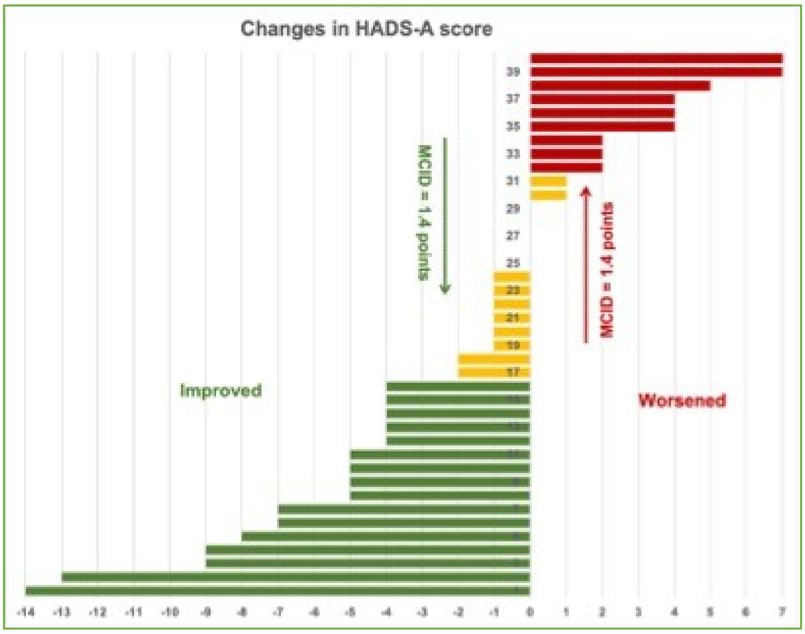

Figure S3: Individual changes in HADS-D

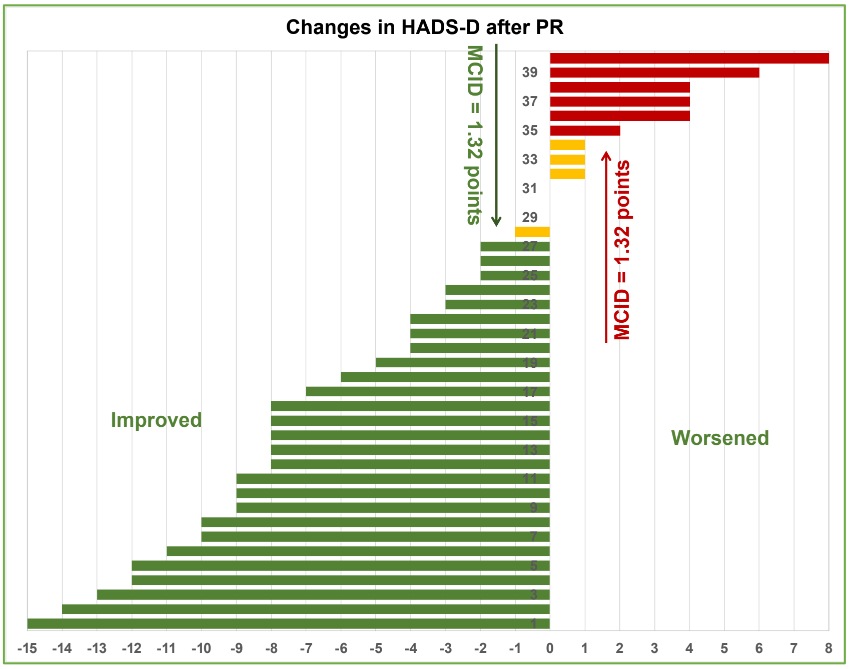

Supplement: Online Supplementary Document [file jogh-15-04002-s001.pdf]
